# Label-Free Impedance Sensing of Aflatoxin B_1_ with Polyaniline Nanofibers/Au Nanoparticle Electrode Array

**DOI:** 10.3390/s18051320

**Published:** 2018-04-24

**Authors:** Ajay Kumar Yagati, Sachin Ganpat Chavan, Changyoon Baek, Min-Ho Lee, Junhong Min

**Affiliations:** School of Integrative Engineering, Chung-Ang University, Heukseok-dong, Dongjak-gu, Seoul 06974, Korea; ajay.yagati@gmail.com (A.K.Y.); sachinchavanmicro@gmail.com (S.G.C.); booguh@naver.com (C.B.)

**Keywords:** polyaniline, Au nanoparticles, aflatoxin B_1_, impedance, microdisk electrodes, immunosensor, antibody, electrochemical impedance spectroscopy (EIS)

## Abstract

Aflatoxin B_1_ (AFB_1_) is produced by the *Aspergillus flavus* and *Aspergillus parasiticus* group of fungi which is most hepatotoxic and hepatocarcinogenic and occurs as a contaminant in a variety of foods. AFB_1_ is mutagenic, teratogenic, and causes immunosuppression in animals and is mostly found in peanuts, corn, and food grains. Therefore, novel methodologies of sensitive and expedient strategy are often required to detect mycotoxins at the lowest level. Herein, we report an electrochemical impedance sensor that selectively detects AFB_1_ at the lowest level by utilizing polyaniline nanofibers (PANI) coated with gold (Au) nanoparticles composite based indium tin oxide (ITO) disk electrodes. The Au-PANI nanocomposites were characterized by scanning electron microscopy (SEM), X-ray diffraction (XRD) spectroscopy, and electrochemical impedance spectroscopy (EIS). The composite electrode exhibited a 14-fold decrement in |Z|_1 Hz_ in comparison with the bare electrode. The Au-PANI acted as an effective sensing platform having high surface area, electrochemical conductivity, and biocompatibility which enabled greater loading deposits of capture antibodies. As a result, the presence of AFB_1_ was screened with high sensitivity and stability by monitoring the changes in impedance magnitude (|Z|) in the presence of a standard iron probe which was target specific and proportional to logarithmic AFB_1_ concentrations (C_AFB1_). The sensor exhibits a linear range 0.1 to 100 ng/mL with a detection limit (3σ) of 0.05 ng/mL and possesses good reproducibility and high selectivity against another fungal mycotoxin, Ochratoxin A (OTA). With regard to the practicability, the proposed sensor was successfully applied to spiked corn samples and proved excellent potential for AFB_1_ detection and development of point-of-care (POC) disease sensing applications.

## 1. Introduction

Aflatoxin B_1_ (AFB_1_) is one of the mycotoxins produced by *Aspergillus flavus* and *Aspergillus parasiticus* species of fungi, mostly found in crops such as grains, maize, peanuts, cereals and are the most potent of hepatocarcinogenic and genotoxic substances [1,2]. Various types of Aflatoxins are categorized such as AFB_1_, AFB_2_, AFG_1_, AFG_2_, and two additional metabolites as AFM_1_ and AFM_2_, with AFB_1_ classified as the most abundant and hazardous [3]. The maximum allowable concentration of aflatoxins set by the United States Food and Drug Administration (parts per billion) is 20 ppb; and according to European Union regulations, the acceptable level of AFB_1_ is 2 ppb in corn used for food and feed [4]. To date, many analytical methods for detection of AFB_1_ have been utilized by extraction and purification steps based on high-performance liquid chromatography (HPLC) [5], thin layer chromatography (TLC) [6], enzyme-linked immunosorbent assay (ELISA) and fluorescence method [7]. Laboratory methodologies for detection of AFB_1_ comprise sample preparation including extraction and isolation, which provides a direct and conclusive response. However, it requires high-level equipment with technical expertise with a lengthy procedure of detection.

Diagnosis of diseases with cheaper, simpler, faster and more sensitive methods are thus of utmost importance for detecting various analytes. In the quest to develop new methodologies, for potential point-of-care application, miniaturized and highly efficient detection systems are crucial. Electrochemical impedance spectroscopy (EIS) based immunosensors are promising systems for obtaining sensitive, multiplexed with portable biosensing configurations. EIS measurements are widely used in the field of electrochemical sensors and biosensors which are performed in the presence of a redox agent, to measure the molecular interactions of electrochemically inactive compounds taking place on the electrode surface for characterization and diagnostics as well as a quantitative detection method. The presence of the redox probe may be undesired as there can be repulsion effects between the upper surface of the adsorbed monolayer and the redox probe, however, other smaller species can reach towards the electrode. Monolayer adsorbed electrodes do not exhibit a perfect capacitive response in the absence of electroactive species at a potential where no surface reaction occurs, rather influenced by the surface roughness of the electrode which can be modeled by constant phase element (CPE) [8] expressed as CPE = {C(*i*ω)^p^}^−1^ where C and p are numerical values, *i* is the imaginary unit, and ω is the angular frequency. Similarly, Nucleic acids such as double helix DNA can be broken into single strand DNA which is oxidizable when exposed to local environments, though this process occurs at relatively high potentials, but there is a strong tendency of oxidized products to be adsorbed on the electrode surface. Therefore, the electrode surface is mostly carbon or other materials instead of gold, or platinum. These adsorptions can be determined by the semicircular high-frequency region of the impedance spectra recorded at half-wave potential and fitted to an RC parallel circuit. The charge transfer resistance (R_ct_) can be utilized to evaluate the standard rate constant of the oxidized species using this expression $R_{\mathrm{ct}}=\left(RT\right)/\left(nF^{2}Ak_{0}c_{\infty }\right)$ where $k_{0}$ is the standard rate constant, A is the area of the electrode, $c_{\infty }$ is the concentration of the electroactive species, *n*, *R*, *t* and *F* have their usual meanings [9]. Recently, various EIS based biosensors utilized the variation in Rct or CPE as a quantifying tool for the detection of food pathogen detection sensors. Microfluidic cell embedded interdigitated electrodes were utilized for the detection of bacteria in food samples [10], printed carbon electrodes were coated with Concanavalin A for bacteria detection in water [11]. Also MoS_2_ nanosheets on Indium tin oxide (ITO) electrodes [12], aptamers on carbon electrode [13], and aptamers on glassy carbon electrodes [14] were demonstrated for the detection of various types of mycotoxins based on the changes in R_ct_. In this sense, EIS based techniques combined with different nanostructures of the receptive surfaces have been applied in the development of AFB_1_ detection. For example, the Wang group [15] proposed an electrochemical immunosensor with AFB_1_ aptamers using a nanocomposite material with differential pulse voltammetry (DPV) as the current output signal technique and obtained a very low detection limit (LOD) of 0.002 fg/mL. The Khan group proposed a method with graphene oxide coupled Au nanoparticles with the DPV method with low LOD of 0.05 ng/mL [16]. Except for the LOD achievements, these systems suffer from limited sensitivity and selectivity with no proper nanostructure formation, certainly leaving room for improvements. Impedance measurements using microdisk electrodes have gained much interest due to their sensitivity, lower detection limits, and high selectivity; however, their significant disadvantage was poor analyte output current. Innovative designs of the electrode were adopted to increase the mass transport towards the electrode [17]. Circular disk electrodes possess uniform flux distribution and provide radial diffusion and the size of the disk influences the overall current density and with shorter RC time constants, and small iR drops make a promising candidate for detecting analyte at low limit of detection [18]. Furthermore, with electrode surface modifications [19,20], the overall electroactive surface area of the electrode will increase, resulting in improved biosensor performance.

Hybrid materials based on metal nanoparticles of gold, silver, iron, and platinum are potential candidates in the development of electrochemical biosensors owing to their biocompatibility, high surface area, excellent conductivity as well as electrocatalytic activity [19]. Also, Polyaniline (PANI) has attracted much attention because of its high specific capacitance due to multi-redox reactions, excellent electronic and thermal properties due to protonation [21], and its low-cost for large quantities. Furthermore, metal nanoparticles conjugated with conducting polymers have been studied extensively due to their synergistic properties inherited from the individual components [22]. The incorporation of nanoparticles in conducting polymer enhance the catalytic property compared to different elements due to the enlarged electroactive surface area and enhanced electrical conductivity of the polymer [23]. Thus, by incorporating these hybrid materials, sensitive, selective, and reliable estimation of analytes at low concentrations could be established [24]. Polyaniline has been utilized for various sensor applications, such as the graphene oxide–polyaniline–Au nanoparticle for cocaine detection [25], the Au nanoparticle–polyaniline nanotube membranes for DNA detection [26], and the polyaniline–iron oxide nanohybrid for catechol detection [27]. However, a Au nanoparticle decorated PANI nanofiber based AFB_1_ sensor in real sample analysis has not yet been reported.

**Figure 1:**
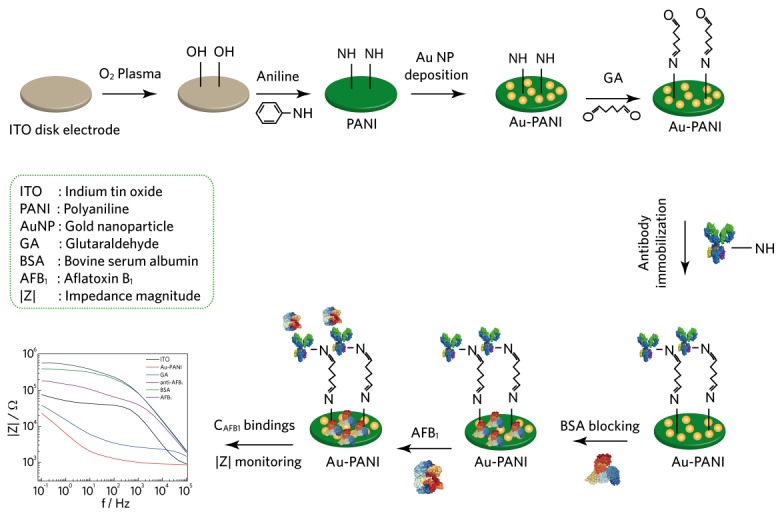
Schematic diagram showing a surface modification of Au–PANI-based disk electrodes for Aflatoxin B_1_ binding with glutaraldehyde (GA) acting as a crosslinking agent to bind anti-AFB_1_ to polyaniline (PANI) nanofibers.

Herein, we report the formation of polyaniline (PANI) nanofibers and gold (Au) nanoparticle composite layers as an efficient biorecognition platform that could selectively detect the AFB_1_ in real samples without the need of target analyte labeling. The immobilization of both PANI and Au on ITO disk electrodes was characterized by EIS in the presence of the redox couple [Fe(CN)_6_]^3−/4−^ with EIS analysis and structurally characterized with the aid of scanning electron microscopy (SEM). For better interpretation of composite electrodes and the sensing process of the experimental data, an equivalent circuit model was developed to explain the interfacial change between the electrode–electrolyte interface. PANI was employed as an active sensing platform, anti-AFB_1_ as the capture antibody, to detect AFB_1_ in corn samples with high sensitivity and selectivity (Figure 1). Considering the advantages of PANI–Au composite materials, the analytical criteria with low detection limit (0.05 ng/mL), linear range (0.1–100 ng/mL) was observed for immunosensor sensor development.

## 2. Experimental Section

### 2.1. Reagents and Materials

Aniline (ACS reagent, ≥99.5%), chloroauric acid (HAuCl_4_), glutaraldehyde solution (50 wt % in H_2_O), anti-aflatoxin B_1_ antibodies (anti-AFB_1_), bovine serum albumin (BSA), potassium ferricyanide and ferrocyanide were purchased from Sigma-Aldrich. Aflatoxin B_1_ (AFB_1_, isolated from *Aspergillus flavus*, ≥98%), and Ochratoxin A (OTA, isolated from *Aspergillus ochraceus*, ≥98%), was purchased from Enzo life sciences, (Seoul, Korea). Hydrochloric acid (1 mol/L) was acquired from Junsei Chemical(Tokyo, Japan). Phosphate-buffered saline (PBS) containing 137 mM NaCl, 2.7 mM KCl, 2 mM KH_2_PO_4_ and 10 mM Na_2_HPO_4_ with pH 7.4, and other aqueous solutions were prepared using Milli-Q water (18 MΩ cm^−1^).

### 2.2. Apparatus

The surface topography of the electrodeposited surfaces was examined by field emission scanning electron microscopy (FESEM) using a Carl Zeiss ΣIGMA. The X-ray diffraction (XRD) analysis was performed using a Rigaku X-ray diffractometer equipped with Cu Kα radiation source of λ = 1.5405 Å using 2θ scanning rate of 0.02° s^−1^. All electrochemical experiments were performed using a CHI 660E workstation (Austin, TX, USA) except when electrochemical impedance spectroscopy (EIS) was carried out, where an IVIUM compactStat was used. All experiments were carried out using a conventional three-electrode system. The ITO disk electrode was used as the working electrode, a large patterned ITO electrode was served as a counter electrode, and the reference electrode was Ag/AgCl (3 M KCl). The EIS measurements were performed at open circuit potential (equilibrium potential), without external biasing in the frequency range of 100 kHz to 0.1 Hz with a signal amplitude of 10 mV in ferricyanide/ferrocyanide ([Fe(CN)_6_]^3−/4−^) redox probe (10 mM; with 0.1 M KCl as a supporting electrolyte). Regarding specifically EIS data, Bode plots were used to plot the obtained spectra showing the frequency response of the electrolyte system and plotting the impedance magnitude (|Z|) against frequency (f). EIS data was fitted using commercially available ZView^®^ software from Scribner Associates (Southern Pines, NC, USA), into the typical Randle’s equivalent circuit that matched the physiochemical process occurring at the hybrid electrode surface.

### 2.3. Preparation of Au-Polyaniline (Au-PANI) on ITO Disk Electrodes

**Figure 2:**
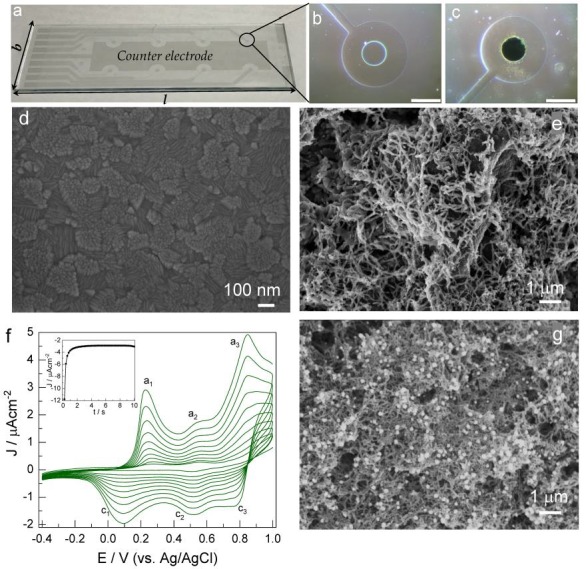
(**a**) Digital picture shows eight working indium tin oxide (ITO) disk electrodes along with large common counter electrode fabricated on glass substrates of dimension 7.5 × 2.5 cm (*l* × *b*); (**b**,**c**) optical images of bare ITO and Au-PANI coated ITO disk electrodes respectively. Scalebar is 500 μm. Scanning electron microscopy of (**d**) bare ITO; (**e**) PANI/ITO electrode; and (**g**) Au-PANI/ITO disk electrode surfaces respectively; (**f**) cyclic voltammogram obtained during potentiodynamic deposition of PANI on ITO electrodes at a sweep rate of 50 mV/s. The highest current peaks belong to the 10th (the last) cycle. The chronoamperometric curve represents the Au deposition process for a duration of 10 s shown in the inset of Figure 2f.

The procedure for the fabrication of ITO (90 Ω/sq) disk electrodes on slide glass (7.5 × 2.5 cm) substrates is presented as follows. Spin-coating an epoxy-based photoresist (SU-8 3005) from Microchem, (Westborough, MA, USA) and developing with standard photolithography procedures, enabled the production of ITO disk-shaped working electrodes (Ø = 500 μm) with a large counter electrode (CE). The development of electrode fabrication resulted in an ITO pattern on a glass substrate consisting of eight working electrodes with a common counter electrode, along with transmission lines and connecting pads (Figure 2a). Before using, ITO electrodes (Figure 2b) were washed with ethanol, and DI water then dried under N_2_ stream.

Aniline polymerization was carried out by conducting cyclic voltammetry experiments with ITO disk electrodes in the potential range of −0.4 to 1 V vs. Ag/AgCl at a scan rate of 50 mV/s for ten cycles in an electrolyte containing aniline (0.25 M) in aqueous HCl (1 M). Change in both CV sweep cycles and scan rate enabled the loading amount of PANI on ITO disk electrodes to be controlled. Chronoamperometry allowed the formation of Au nanoparticles on PANI coated ITO electrodes in an electrolyte solution (HAuCl_4_·3H_2_O; 1 mM) at a deposition voltage of −0.9 V vs. Ag/AgCl for the duration of 10 s. After each deposition, the resultant modified electrodes were washed with DI water and dried under a N_2_ stream (Figure 2c).

### 2.4. Surface Engineering of Impedimetric AFB_1_ Sensor

The capture antibody (anti-AFB_1_) was immobilized on the PANI-Au/ITO electrode surface with glutaraldehyde linking between the residual amine (NH_2_) groups, between PANI and antibody. Specifically, the PANI-Au/ITO electrodes were activated by immersion in 1% (*v*/*v*) solution for 90 min at room temperature. Following rinsing with water, 20 μL of 100 µg/mL anti-AFB_1_ was spread onto the resulting electrode surface to allow incubation at 4 °C in an ice bath for 2 h. After rinsing, with a copious amount of PBS buffer to remove unbound anti-AFB_1_ molecules on the Au-PANI/ITO electrode surface, 20 μL of 1% (*w*/*v*) BSA solution was dropped onto the modified electrodes at room temperature for 90 min to block the nonspecific binding sites. The modified electrode was incubated with different concentrations of AFB_1_ antigen samples for 1 h at room temperature. Finally, the developed immunosensor was washed with water and stored at 4 °C for further use. To test the specificity of the developed immunosensor, OTA (100 ng/mL) and corn samples, both pure and spiked with AFB_1_ (1 ng/mL), were used as interfering proteins.

### 2.5. Aflatoxin B_1_ Spiking Test for Real Sample Analysis

Different concentrations of AFB_1_ were spiked in pure corn powder for the development of the real sample analysis system. The concentrations of AFB_1_ with 0.1, 1, and 100 ng/mL (200 μL) were added to 500 mg of corn powder; then the dried spiked corn powder was mixed with 2 mL of methanol/PBS (volume ratio, 90:10). The mixture was shaken for 30 min and centrifuged at 3000 rpm for 10 min. Finally, 1 mL supernatant was collected and diluted with (deoxygenated) PB filtered through 0.2 μm pore size membrane filters.

## 3. Results and Discussion

### 3.1. Formation and SEM Characterization of Au-PANI Nanofibers on ITO Disk Electrode

The composite microstructures of Au-PANI formed onto the ITO disk electrodes through electropolymerization were investigated by FESEM analysis. The PANI electrodeposited surface showed uniform nanofibers entangled over the entire surface (Figure 2), thus, this indicates that PANI fibers were formed without any degradation with ten cycles during electropolymerization, a further higher number of cycles results in aggregated and uneven PANI structures. After electrodeposition of Au on the PANI electrodes, SEM analysis revealed that Au nanoparticles were coupled to PANI nanofibers and evenly distributed having a size ranging from 80 to 100 nm making a composite structure of Au-PANI on the ITO disk electrode as shown in Figure 2g. The potentiodynamic approach enabled the polymerization of PANI nanofibers on ITO disk electrodes, and corresponding cyclic voltammogram (CV) were recorded for ten continuous cycles, as shown in (Figure 2f). The CV peaks corresponding to a_1_-c_1_ at 0.2 V (Figure 2f) were attributed to a quasi-reversible reaction for the formation and reduction of the leuco-emeraldine form of PANI to proto-emeraldine [28]. Further, the peaks a_2_-c_2_ and a_3_-c_3_ were attributed to oxidation of the latter to emeraldine and subsequent oxidation to nigraniline respectively.

The following sequence is proposed to describe the growth of the films on the ITO disk electrode [29]:

(1) $${\left(\mathrm{ANI}\right)}_{n}^{\mathrm{red}}\to {\left(\mathrm{ANI}\right)}_{n}^{\mathrm{ox}}+{\mathrm{ne}}^{-}\;$$

$${\left(\mathrm{ANI}\right)}_{n}^{\mathrm{ox}}+\mathrm{ANI}\to \;\to \;\to {\left(\mathrm{ANI}\right)}_{n-1}^{\mathrm{ox}}{\left(\mathrm{ANI}\right)}_{2}^{\mathrm{red}}$$

Here ANI is anline, ${\left(\mathrm{ANI}\right)}_{n}^{\mathrm{red}}$ is the entirely reduced form, and ${\left(\mathrm{ANI}\right)}_{n}^{\mathrm{ox}}$ is a fully oxidized form of PANI, i.e., diradical dication. The diradical dication formed on the PANI chain terminal undergoes a coupling reaction with an aniline molecule while accepting electrons from it. After one cycle of this process, *n* becomes *n* + 1, and the PANI film grows and retains the highest oxidation state. Subsequently, the polymer chain is ready to repeat the next cycle termed as an autocatalytic mechanism. Similarly, the Au deposition on the PANI coated ITO electrodes was involved in the reduction of ${\mathrm{AuCl}}_{4}^{-}$ that forms well distributed Au nanoparticles on the surface. The chronoamperometric deposition resulted in a rapid increase in current density during the first 2 s and reached a fixed value for the remaining period of deposition (inset Figure 2f). During the initial period of deposition, the observed decrease in current was due to the charge transfer controlled flow resulting due to the reduction in the concentration of ${\mathrm{AuCl}}_{4}^{-}$ at the electrode surface. Later, the mass transports by diffusion led to the depletion of ${\mathrm{AuCl}}_{4}^{-}$ ions to determine the overall reduction reaction. The growth of the nanoparticle size determines this mass transport of ions. Also, with an increase in diffusion layer thickness, there is current decay which follows Cottrell’s law due to the decrease in a concentration gradient of ions at the PANI electrode surface.

### 3.2. Electrochemical Impedance Characterization of the Au-PANI Electrode

**Figure 3:**
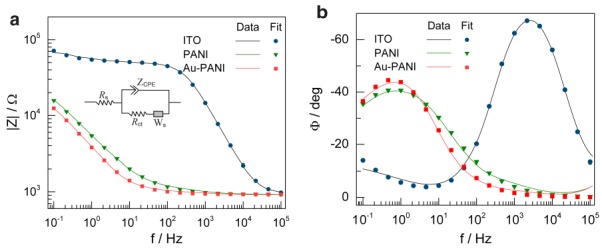
Bode plot shows (**a**) |Z| vs. f and (**b**) Φ vs. f for bare ITO, PANI/ITO, and Au-PANI/ITO electrodes respectively. Inset of figure (**a**) shows the equivalent model circuit used to extrapolate the parameters through impedance fitting analysis.

Electrochemical impedance spectroscopy (EIS) was employed to characterize the Au-PANI/ITO electrode system under study. A small amplitude AC signal over a wide frequency range was used to measure the charge transport properties and complex impedance characteristics to characterize the electrodes after each deposition process for the fabrication of the Au-PANI/ITO electrode. Bode plots were recorded for bare, PANI and Au-PANI-coated on an ITO disk electrode in a [Fe(CN)_6_]^3−/4−^ redox probe respectively (Figure 3) and an equivalent circuit model was fitted to the experimental data. The fitting analysis revealed that solution resistance (Rs) possessed least changes on all electrodes indicating uniform current distribution on all electrodes, however, bare ITO possessed the highest charge transfer resistance (Rct) value of 54.8 kΩ with constant phase element (CPE-T) value of 0.027 µF with (CPE-P) = 0.893. A decrease in Rct value to 45.5 kΩ with a significant increase in the CPE-T value of 67.02 µF was observed with the electrochemical polymerization of PANI on the ITO disk electrode. The equivalent circuit elements from the fitted impedance data are given in Table 1. The decrease in the Rct value was due to the conductive PANI film with a significant enhancement electroactive surface area on the ITO disk electrode which allows increased mass transport of [Fe(CN)_6_]^3−/4−^ ions towards the electrode. With the subsequent modification of Au nanoparticles on the PANI deposited ITO; the Rct was further decreased to 27.15 kΩ having CPE-T of 84.69 µF. This is attributed to the large surface area of the nanocomposites and the synergetic effect of the binary composites. Therefore, Au-PANI coated electrodes were adopted for further studies and utilized for the AFB_1_ detection sensor.

### 3.3. Optical Characterization of Au-PANI Electrodes

**Figure 4:**
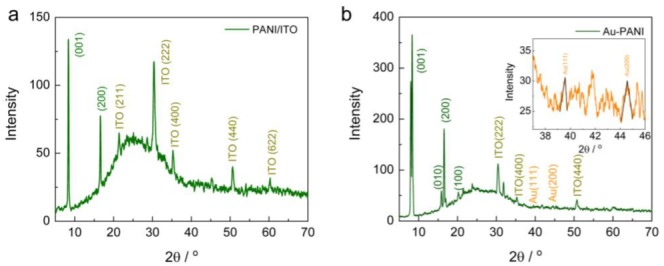
X-ray diffraction (XRD) patterns obtained for (**a**) PANI/ITO and (**b**) Au-PANI/ITO electrodes respectively; inset of Figure 4b shows the magnified view of the XRD peaks of Au nanoparticles in the range from 37–46 deg.

The XRD analyses on both PANI and Au-PANI deposited electrode surfaces were examined to observe the structure of the nanocomposite materials as shown in Figure 4a,b. The PANI-coated ITO showed peaks at 8.3° and 16.58°, which match the diffraction patterns for (001) and (200) orientations of PANI with a d spacing of 4.593 Å and 3.461 Å, respectively. This confirms that the PANI deposited electrodes were shown to be crystalline and were well formed on the ITO electrodes. Furthermore, Au-PANI coated electrodes exhibited two additional peaks at 38.2° and 44.6° corresponding to Au (111) and Au (200) along with PANI peaks which are in good agreement with JCPDS card file #65-2870. The intensity of PANI peaks dominated the Au because of having the larger surface area; overall the Au-PANI composite electrode was crystalline and well formed on the ITO surface.

### 3.4. EIS Characterization of the Au-PANI Layer for Surface Immobilization

**Figure 5:**
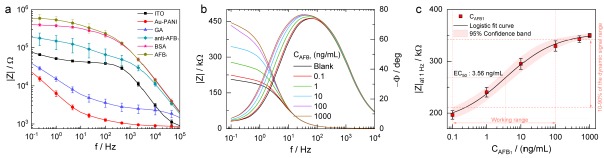
(**a**) Bode plots obtained after various steps of the surface modification process for (■) ITO; (●) Au-PANI; (▲) glutaraldehyde (GA); (◆) anti-AFB_1_; (⋆) for BSA and (⬟) for AFB_1_ (100 ng/mL) for developing the aflatoxin detection sensor. Data points indicate the mean ± SD of four replicated impedance measurements; (**b**) Impedance magnitude measured from different concentration of aflatoxin B_1_ (C_AFB1_) in phosphate buffer saline (PBS) measured from blank, 0.1 to 1000 ng/mL over the frequency range of 1 to 10^6^ Hz; (**c**) Calibration curve with 95% confidence bands for AFB_1_ sensor obtained at |Z|_at 1 Hz_ for various C_AFB1_. Data points indicate the mean ± SD of four replicated impedance measurements, and the fit curve represents the logistic four-parameter regression.

EIS is a useful analytical tool for understanding the electrode-electrolyte interface and for examining the surface modification of the electrode by observing the Bode or Nyquist diagrams in the [Fe(CN)_6_]^3−/4−^ redox probe. Therefore, the Bode plots were obtained for all steps to construct the AFB_1_ recruiting surface and were characterized as shown in Figure 5a. The bare ITO electrode showed typical characteristics, where R_s_ dominated the high-frequency region, the intermediate-frequency reflected the double-layer capacitance (C_dl_), and the lower-frequency part revealed the charge transfer resistance (R_ct_). However, the Au-PANI-coated electrode showed lower values in impedance, where R_s_ was dominant from 1 to 100 kHz and from 1 kHz to 0.1 Hz, the CPE resulted. The overall decrease in |Z| is due to the high surface area of the Au-PANI nanocomposite surface. However, subsequent treatment with GA inhibited the movement of anionic redox indicator ions towards the electrode; thus, |Z| increased, and only the CPE and R_ct_ appeared. It can be observed that |Z| (R_ct_, the transducer parameter) increases together with the bindings of anti-AFB_1_, blocking with BSA, and AFB_1_ due to an increase in the steric blockage of the surface caused by the modifications on the electrode, resulting in a suppression of the electrode transfer kinetics. The accuracy of the AFB_1_ biosensors was tested by performing measurements for |Z| vs. f using three different electrodes, towards relative standard deviation (RSD) for the measurements of the AFB_1_ detection sensor.

### 3.5. Impedance-Based AFB_1_ Sensor Characterization

Under optimized conditions, the analytical performance of the sensor was tested by capturing AFB_1_ of various concentrations (0.1 to 1000 ng/mL) by observing the changes in impedance. The measured |Z| were recorded and found to increase with increasing AFB_1_ concentration, as shown in Figure 5b. A significant change in |Z| was observed from 1 to 10^3^ Hz owing to the interaction of AFB_1_; thus, |Z|_at 1 Hz_ was recorded after each addition of AFB_1_. To determine the sensitivity and detection limit of the developed sensor, a calibration curve method was utilized for the change in impedance magnitude for C_AFB1_ in PBS as shown in Figure 5c. The calibration curve was fitted using a standard 4-parameter logistic equation according to the following formula [8]

(2) $$y=A_{2}+\left[\frac{\left(A_{1}-A_{2}\right)}{1+{\left(\frac{x}{{\mathrm{EC}}_{50}}\right)}^{b}}\right]$$

Here, *y* is impedance magnitude (|Z|), *x* is a concentration of AFB_1_ (C_AFB1_) in ng/mL, A_1_ and A_2_ are the minima and maximum analytical response, *b* represents slope at the inflection point, and EC_50_ is the concentration leading to 50% of the maximum signal, respectively. The best fit values of the experimental data measured in PBS were *y* = 354.48 + (181.91 − 354.48)/(1 + (*x*/3.56)^0.59^). This fit showed a correlation coefficient *R*^2^ = 0.998, an EC_50_ of 3.56 ng/mL and a detection limit of 0.05 ng/mL. The limit of detection was calculated as X + 3σ, where X was the average value of EIS for blank signals (obtained in the absence of antigen) and σ was the known standard deviation of EIS for the blank signal [30]. The detection limit was lower than the previous reported AFB_1_ sensors developed by different modified electrodes shown in Table 2. Further, the developed sensor showed a linear range of 0.1–100 ng/mL.

Furthermore, the measurements of the coefficient of variation (CV) from intra-assay and inter-assay testing enabled the evaluation of the reproducibility of the developed sensor. Impedance measurements of AFB_1_ with 100 ng/mL in four replicate measurements ensured the precision measurements for intra and interassay variation analysis for BSA/anti-AFB_1_/GA/Au-PANI/ITO electrodes. The intra-assay and inter-assay RSDs were both below 5%. Assay results indicate that the developed sensor possesses good reproducibility with acceptable precision. To further validate the reliability of the developed sensing system to real-time analysis, the recovery and accuracy method in corn samples was evaluated, and the results are presented in Table 3. The AFB_1_ spiked in pure corn samples were subjected to the impedance measurement system, and the results indicated that the level of recovery of the respective 0.1, 1 and 100 ng/mL spiked samples were 98.5%. 108.7%, and 106.3% with an RSD less than 6%, indicating the excellent accuracy of the proposed immunoassay for quantitative detection of AFB_1_ based Au-PANI composite electrodes.

### 3.6. Selectivity, Stability, and Reproducibility of the Developed AFB_1_ Sensor

**Figure 6:**
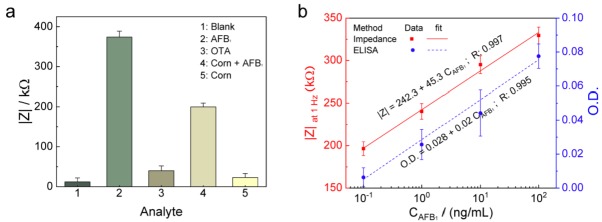
(**a**) Selectivity of the developed sensor demonstrated by impedance measurements with various types of the analyte to examine the cross-reactivity of the AFB_1_ sensor; (**b**) impedance change and optical density (O.D.) obtained by ELISA versus AFB_1_ concentrations (0.1 to 100 ng/mL).

The selectivity of the developed sensor was evaluated by application of OTA (100 ng/mL), corn, AFB_1_ (100 ng/mL) and AFB_1_ spiked corn samples (1 ng/mL). No significant changes were observed when OTA and corn samples were applied to the sensing system. However, the vast change in |Z| was observed for AFB_1_ and AFB_1_ spiked corn samples. These findings confirm that the developed sensor is selective to detect AFB_1_ and is not affected by the presence of other contaminating mycotoxins of similar molecular weight (Figure 6a). The reusability of the sensor was evaluated by observing the changes in Nyquist plots for various modification processes in the development of AFB_1_ detection. The impedance sensor was incubated with 0.1 M glycine-HCl at pH 3.0 for 10 min and subsequently washed with DI water. The resulting impedance measurements revealed that the AFB_1_ along with some anti-AFB_1_ antibodies was eluted, which enabled the possibility of reutilizing the sensor, however at present, the sensor is for one-time use only. Also, the operational stability is one of the primary factors for evaluating the performance of the developed sensor for practical application. The sensor retained its impedance response for a storage period of 12 days in PBS (10 mM, pH 7.0) when kept at −4 °C. The results show that no apparent denaturation occurred, and the antibody-coated Au-PANI hybrid electrodes provided an adequate biocompatible environment to preserve the activity towards AFB_1_ detection. It is expected that the glutaraldehyde interaction between the amine-terminated PANI and the primary −NH_2_ groups of the antibodies prevented the desorption of AFB_1_ antibodies. Furthermore, a comparative study of the developed immunosensor with enzyme-linked immunosorbent assay (ELISA) was performed (Figure 6b). The immunosensor exhibited linearity that is comparable to that for conventional methods (ELISA) and had a detection limit suitable for on-site monitoring. The simplicity of the method should also make it suitable for use in the detection of many other toxins and environmental pollutants. Thus, the main advantages of the developed impedance-based AFB_1_ sensor over other methods are that it is label-free and inexpensive and requires minimal sample preparation for sensitive detection of the analyte signal. Recently many impedance based biosensors have been reported, however, their commercial applications have been hindered due to several technical limitations such as limitations in detecting small analytes, the complex nature of impedance detection, stability of biomaterial immobilization, and prone to nonspecific adsorptions. Though the present approach overcomes the limitations such as stability of the electrode and sensing various analytes, it still requires extensive study and impedance circuitry to be developed for real point-of-care (POC) applications.

## 4. Conclusions

In conclusion, a simple electrochemical immunoassay of high sensitivity and efficiency was developed for the detection of AFB_1_ by a robust covalent coupling between antibodies and Au-PANI through GA linker. The Au-PANI has not only excellent conductivity and larger surface area but also provided abundant binding sites for the conjugation of anti-AFB_1_. PANI nanofibers were evenly distributed, with Au nanoparticles decorated on it, offering enhanced ionic and electronic transport in comparison with the bare disk electrode that resulted in a 14-fold decrement in |Z| at 1 Hz. The structural, optical, and electrochemical properties were evaluated through analytical tools to examine the morphology and conductivity of Au-PANI electrodes. A good linear relationship between |Z|_at 1 Hz_ and C_AFB1_ was obtained in the range of 0.1–100 ng/mL with a LOD of 0.05 ng/mL. The convenient operation and high sensitivity of the proposed immunoassay could result in it becoming a promising tool in the detection of AFB_1_ for real application in the field of food safety detection.

## Figures and Tables

**Table 1:** Values of the equivalent circuit elements obtained by fitting the experimental results of various electrode modifications for Bode plots represented in Figure 3a and the corresponding relative errors using the equivalent circuit model consisting of the solution resistance (Rs), the constant phase element for the electrode interfacial impedance (CPE) and the charge transfer resistance (Rct).

Electrode	R_S_ [Ω]	CPE		Rct [Ω]
		T [×10^−9^ Ω^−1^·s^P^]	P	
Bare-ITO	918.7 ± 30.01	0.027 ± 0.005	0.893 ± 0.021	54,852 ± 1803.3
PANI-ITO	925.2 ± 20.83	67.02 ± 4.911	0.585 ± 0.021	45,597 ± 129.8
Au-PANI-ITO	922.7 ± 18.84	84.69 ± 5.99	0.680 ± 0.025	27,154 ± 571.7

**Table 2:** The comparative analytical performance of the sensor with reported literature for AFB_1_ detection.

Sensing Technique	Transducing Matrix	Linear Range (ng/mL)	Limit of Detection (ng/mL)	Reference
EIS, CV	Aptamers on dendrimers	0.1–10	0.4	[31]
Bio imprinting	Au electrode	1–1000	0.00197	[32]
EIS	AFB_1_/BSA/Au	0.08–100	0.05	[33]
EIS	SPIM ^a^ Au electrode	-NA-	5.0	[34]
EIS	Aptamer/GCE ^b^	0.1–100	0.05	[35]
EIS	MWCNTs ^c^/RTIL ^d^	0.1–10	0.03	[36]
EIS	Ab ^e^-SGIL ^f^-GCE	0.1–10	0.01	[37]
EIS	Au-CD-trodes ^g^	1.56–31.2	0.11	[38]
EIS	GO ^h^/Au	0.5–5	0.23	[39]
EIS	PPy ^i^/PPa ^j^/rGO ^k^	0.0001–0.01	0.0001	[40]
EIS	PoPD ^l^/3DNEEs ^m^	0.04–8	0.019	[41]
ELISA ^n^	monoclonal antibodies	0.1–10	0.05	[42]
ELISA	monoclonal antibody	0.05–10	0.036	[43]
HPLC ^o^	immunoaffinity columns	4–20	3.5	[44]
EIS	Ab/BSA/Au-PANI/ITO	0.1–100	0.05	This work

^a^ SPIM: Screen-printed interdigitated microelectrodes; ^b^ GCE: Glassy carbon electrode; ^c^ MWCNTs: Multi-wall carbon nanotubes; ^d^ RTIL: Room temperature ionic liquid; ^e^ Ab: Aflatoxin antibody; ^f^ SGIL: Silica gel ionic liquid; ^g^ CD-trodes: (electrodes obtained from recordable compact disks); ^h^ GO: Graphene oxide; ^i^ PPy: Polypyrrole; ^j^ PPa: Pyrrolepropylic acid; ^k^ rGO: Reduced graphene oxide; ^l^ PoPD: Poly(*o*-phenylenediamine); ^m^ 3DNEEs: gold three dimensional nanoelectrode ensembles; ^n^ ELISA: Enzyme-linked immunosorbent assay; ^o^ HPLC: High-performance liquid chromatography.

**Table 3:** Analysis results for determination of AFB_1_ in the spiked corn sample.

Sample	Added (ng/mL)	Found (ng/mL)	Recovery (%)	RSD (%) (*n* = 3)
Corn	0.1	0.094	94.5	5.25
	1	0.98	98.2	2.56
	100	103.7	103.7	3.06
